# HOXD9/APOC1 axis promotes macrophage M1 polarization to exacerbate diabetic kidney disease progression through activating NF-κB signaling pathway

**DOI:** 10.1186/s41065-024-00345-9

**Published:** 2024-11-07

**Authors:** Ya Feng, Yalan Zhang, Fang Gao, Miaomiao Liu, Yangyan Luo

**Affiliations:** 1 Department of Nephrology, The First Affiliated Hospital of Chengdu Medical College, No. 278, Baoguang Avenue, Xindu District, Chengdu, 610500 China; 2 School of Clinical Medicine, Chengdu Medical College, Chengdu, 610500 China

**Keywords:** Diabetic kidney disease, Exosomes, HOXD9, APOC1, Macrophage M1 polarization

## Abstract

**Background:**

Diabetic kidney disease (DKD) is a complication caused by end-stage diabetes mellitus and usually results in glomerular podocyte injury. Exosomes are important for intercellular information exchange. However, the effect of podocyte exosomes on DKD has not been elucidated.

**Methods:**

GEO, PROMO, and GSE1009 databases were used to identify the gene APOC1 and transcription factor HOXD9. qRT-PCR, western blot, and transmission electron microscopy (TEM) were investigated to confirm APOC1 change in high glucose-treated podocytes and exosomes. Flow cytometry, immunofluorescence, qPCR, immunoblotting, wound healing, Transwell invasion assays, dual luciferase assay, and ChIP-PCR assay were performed to detect the effect of APOC1 and HOXD9 on macrophage polarization in high glucose-treated podocytes and exosomes. qRT-PCR and immunoblotting assays were employed to assess the impact of APOC1 knockdown on the M1 polarization of macrophages in response to liraglutide treatment.

**Results:**

The results suggested that the expression of APOC1 in human podocytes (HPC) and exosomes was elevated. High glucose-treated HPC exosomes promoted macrophage M1-type polarization, which was reversed by adding sh-APOC1. Afterward, HOXD9 was identified as a potential transcription factor for APOC1. Knockdown of HOXD9 led to macrophage M2 polarization, and overexpression of APOC1 polarized macrophage M1. In addition, enhanced p65 phosphorylation verified that HOXD9/APOC1 induced macrophage M1-type polarization by activating the NF-κB signaling pathway. Knocking down APOC1 enhanced the inhibitory effect of liraglutide on macrophage M1 polarization.

**Conclusion:**

Our findings highlighted that HOXD9/APOC1 was a key player in causing podocyte injury in diabetic kidney disease and led to macrophage M1 polarization through the NF-κB signaling pathway.

**Supplementary Information:**

The online version contains supplementary material available at 10.1186/s41065-024-00345-9.

## Introduction

Diabetes is on the rise globally as the lifestyle habits of people change. According to research from the International Diabetes Federation (IDF), the number of adults with diabetes worldwide reached 537 million in 2021, a 16% increase compared to 2019, and the number of people with diabetes is expected to reach 784 million in 2045 [1–3]. Diabetic kidney disease (DKD) is one of the most common and serious chronic microvascular complications of diabetes and has become the leading cause of end-stage renal disease. A rise in glucose causes an increase in glycolysis and the synthesis of metabolites, which via multiple mechanisms promote oxidative stress, inflammation, and fibrosis [4]. The primary glomerulopathy caused by DKD includes albuminuria, glomerular hypertrophy, podocyte damage, and reduced glomerular filtration rate (GFR). Diffuse and nodular tethered dilatation and thickening of the glomerular basement membrane (GBM) are subsequent effects of DKD [3, 5].

The involvement of exosomes in the pathophysiologic development of DKD has been reported. Exosomes are nanosized extracellular vesicles containing substances such as DNA, RNA, lipids, proteins, and inflammatory factors secreted by almost all cells [6, 7]. Previous studies indicated that FOXO1 was phosphorylated and inactivated in DKD, leading to downregulation of RAB27B and decreased exosome secretion [8]. The cell types of glomeruli include podocytes, endothelial cells, and thylakoid cells. Additionally, Ding et al. found that Sirtuin1 (Sirt1) was significantly downregulated and exosome secretion was increased in podocytes of DN, leading to the inhibition of lysosomal acidification [9]. Recent research reported that high glucose-stimulated proximal tubular epithelial cells (PTEC) secrete the exosome miR-92a-1-5p, which decreased reticulin 3 expression and caused endoplasmic reticulum (ER) stress [10]. Therefore, it is crucial to explore podocyte exosomes in diabetic kidney disease.

Apolipoprotein C1 (APOC1) is a single-chain protein belonging to the apolipoprotein C family. APOC1 is initially synthesized in the endoplasmic reticulum and has a molecular weight of 9.3 kDa. Mature apolipoproteins, the smallest apolipoproteins with molecular weights of 6.6 kDa, are produced when it is sheared [11]. APOC1 plays an important role in lipid metabolism and immune inflammation. Likewise, APOC1 is closely related to the progression of many diseases such as malignancy and Alzheimer’s disease [12, 13]. In 2020, Cui et al. identified APOC1 as a novel diagnostic marker for clear cell renal cell carcinoma [14]. Furthermore, another study revealed that APOC1 is a biomarker for DKD through machine learning algorithms and experiments [15]. APOC1 promotes breast cancer progression through the EMT and MAPK/JNK pathways [16]. However, changes in APOC1 in podocyte exosomes and the genes that regulate APOC1 remain unknown.

Inflammation plays a major role in the development of DKD [17, 18]. Inflammation-related diseases tend to result in alterations to macrophages [19]. Studies have shown that macrophages differentiate into M1 and M2 types upon external stimuli, with M1-type macrophages appearing in the pre-inflammatory phase and promoting inflammation. While M2-types appear mainly in the middle and late phases of inflammation, mainly mitigating the inflammatory effects [20]. Recent studies have illuminated that APOC1 could be detected in the kidneys of diabetic patients, suggesting that APOC1 may be involved in glomerulosclerosis. Further experimentation revealed that APOC1 facilitated the release of inflammatory mediators through the augmentation of cytokine signaling in macrophages. This intensified inflammatory reaction may precipitate additional injury to mesangial cells, consequently accelerating the development of glomerulosclerosis [21]. Another study reported that APOC1 could reduce iron death in non-small cell lung cancer (NSCLC) patients through the NRF2/HO-1 pathway, which in turn inhibited the conversion of M2 to M1 macrophages for anti-PD-1 immunotherapy for NSCLC [22]. Similarly, a study by Yu and colleagues selected genes related to IgA nephropathy (IgAN) through the GSE35487 database, and in vivo and in vitro experiments showed that the expression of APOC1 was elevated in IgAN. Subsequently, they identified that IgAN renal fibrosis could be ameliorated by knocking down APOC1 to inhibit the NF-κB pathway [15]. The collective research underscores the complex and sometimes contradictory roles of APOC1 in different disease states, highlighting its importance as a target for further investigation in kidney disease.

In the realm of diabetes therapeutics, glucagon-like peptide-1 receptor agonists (GLP-1RA) represents a significant classe of medications [23]. GLP-1RA enhances insulin secretion and suppresses glucagon release by stimulating the GLP-1 receptor. It also promotes satiety, slows gastric emptying, and reduces food intake, thereby lowering blood glucose levels. Furthermore, GLP-1RA may mitigate atherosclerosis by improving inflammatory markers. Its anti-inflammatory and antioxidant effects may reduce albuminuria, ameliorate glomerular hyperfiltration, and improve endothelial function, thus exerting a protective effect on the kidneys [24]. As a GLP-1 receptor agonist, liraglutide has demonstrated potential therapeutic efficacy in the treatment of DKD. Studies have shown that rat mesangial cells (MCs) cultured under high-glucose conditions and subsequently treated with liraglutide exhibit a significant reduction in the activation of the TLR4/MYD88/NF-κB signaling pathway, which is typically induced by elevated glucose levels. This treatment also results in decreased expression of extracellular matrix (ECM)-related proteins and inflammatory factors [25]. Furthermore, liraglutide is recognized for its renal protective effects mediated through the SIRT1/TXNIP pathway [26]. However, the potential role of APOC1 in mediating the effects of liraglutide remains to be elucidated.

In this study, we investigated the effect of APOC1 in podocyte exosomes on DKD and also identified HOXD9, a transcription factor that affects APOC1, followed by evaluating whether HOXD9/APOC1 affects the progression of DKD through activation of the NF-κB signaling pathway. This study aims to provide new insights into the pathogenesis of DKD and to identify new targets for the treatment of DKD.

## Materials and methods

### Bioinformatics analysis

APOC1 expression in patients with diabetic kidney disease was screened using the GEO database (https://www.ncbi.nlm.nih.gov/geo/) as well as GSE microarrays 1009. The PROMO database was used to predict potential transcription factors for APOC1, followed by further focus on the expression of the screened transcription factors using GSE1009.

### Cell culture

Human podocytes (HPC) were purchased from Immocell company (Xiamen, China). HPC was cultured in DMEM medium containing 10% fetal bovine serum (FBS, Gibco, Waltham, USA) and 1% penicillin/streptomycin (Gibco, Waltham, USA), and placed into a 37 °C, 5% CO_2_ cell culture incubator for 2–3 days. Passaging was performed when the cells grew to 80–90% concentration. To assess the effect of high glucose on HPC, cells were incubated with a medium containing 5 mM glucose (physiologic glucose concentration) or 30 mM glucose (HG concentration) for 48 h.

Human monocyte THP-1 was purchased from the Shanghai Cell Bank of the Chinese Academy of Sciences (Shanghai, China). THP-1 cells were cultured in RPMI-1640 medium (Gibco, Waltham, USA) containing 10% fetal bovine serum and 1% penicillin/streptomycin, after which they were placed in the culture at 37 °C with 5% CO_2_.

### Exosome isolation

After HPC was treated according to the above procedure, the podoplanar exosomes were isolated by differential centrifugation. The supernatant was first collected and centrifuged at a low speed of 300 × g for 10 min at 4 °C. The precipitate was discarded and the supernatant was retained. Subsequently, centrifuge at 2,000 × g for 20 min and retained the supernatant. This was followed by centrifugation at 10,000 × g for 30 min to take the supernatant. Finally, 100,000 × g ultracentrifugation for 90 min was performed and the precipitate was retained. The resulting precipitate was resuspended in 0.2 μm filtered phosphate-buffered saline (PBS) and used for transmission electron microscopy (TEM) analysis. Alternatively, they were placed in DMEM for processing, lysed for protein analysis, or harvested and stored at -80 °C for further processing.

### Macrophage differentiation and cell co-culture

THP-1 was induced into M0 macrophages by adding 100 ng/ml of fopperol 12-myristate 13-acetate (PMA, Santa Cruz Biotechnology, Dallas, USA) to THP-1 cells, followed by incubation for 48 h at 37 °C in a 5% CO_2_ incubator. The cells were washed twice with PBS and incubated in RPMI-1640 medium.

To investigate whether exosomes secreted by HG-stimulated podocytes were involved in the polarization of M0 macrophages, we used Transwell chambers (membrane pore size of 0.3 μm), thus ensuring that podocytes were co-cultured with M0 macrophages without direct cell-to-cell contact. The M0 macrophage suspension was inoculated in the lower chamber of the Transwell, and the HPC exosomes were inoculated in the upper chamber of the Transwell for 48 h.

### qRT-PCR

Total RNA from HPC or HPC exosomes was first extracted using Trizol reagent (Takara, Tokyo, Japan). Secondly, real-time qPCR was performed using SYBR Green PCR MASTER MIX (Applied Biosystems) in the ABI Prism 6000 Sequence Detection System (Applied Biosystems). Quantitative polymerase chain reaction results were obtained by using the Livak method (2^−∆∆Ct^) expressed as fold change mean ± standard error of the mean (Sm). Primer sequences used for qRT-PCR are listed below. Primers pairs used in the present study were as follows: APOC1 forward, 5’-CTGAGTGGGGAAAGGGACTA-3’ and reverse, 5’-GGAGGGGCACTCTCTCAATC-3’. GAPDH as an internal reference.

### Transmission electron microscopy (TEM)

The exosome particle solution was coated on a Formvar/carbon coated 200 mesh copper electron microscope grid, stained with uranyl acetate for 5 min at room temperature, and then observed in a transmission electron microscope (Hitachi H7500 TEM, Tokyo, Japan) at 80 kV.

### Flow cytometry

THP-1 cells were incubated with fluorescently labeled antibodies for 30 min in the dark and then washed with FACS buffer (BSA in 2% PBS). Cell ratio analysis was performed using FlowJo software.

### Immunofluorescence staining

Dissolve PKH67 in anhydrous ethanol or DMSO to prepare a stock solution of 4 mM, add PKH67 to the tube containing exosomes to dilute to 2 µM, mix well so that the exosomes are in full contact with the dye, and stain for 15–20 min away from the light. After completion of the staining, the reaction was terminated by adding serum and the unbound dye and serum were removed using an ultracentrifuge, and the washing was repeated three times by centrifugation.

### Cell transfection

To knockdown APOC1 and HOXD9 and overexpress APOC1, full cDNA sequences of APOC1 and HOXD9 were synthesized and inserted into pLVX-puro vector to generate pLVX-APOC1 and pLVX-HOXD9, and the empty vector was used as a negative control. Western blotting and qRT-PCR were used to confirm the transfection effects described above. The pLVX vectors containing shRNA sequences of APOC1 or HOXD9 were constructed by Gemma Genetics Suzhou. Lentiviruses containing (sh-APOC1, sh-HOXD9, sh-NC, OE-APOC1, and OE-NC) vectors were purchased from Gemma Gene Suzhou. Lentiviral infection was performed in HPC exosomes. Afterward, puromycin (4 µg/ml) was added to generate a stable transducer library.

### Transwell invasion assay

To assess the invasive ability of M0 macrophage cells co-cultured with HPC exosomes, 80 µL of Matrigel (Corning) was pre-coated in the upper part of Transwell chambers with a pore size of 8 μm and allowed to solidify in an incubator at 37˚C for 30 min. Cells (4 × 10^5^) resuspended in serum-free RPMI-1640 were inoculated into the upper chamber. Suspension with serum-free RPMI-1640 was then inoculated into the upper chamber, complete medium with 10% FBS was added to the lower 24-well plate. The cells were incubated in an incubator at 37˚C for 24 h. After removal, the cells were fixed with 4% paraformaldehyde for 30 min at room temperature and then stained with 0.1% violet crystal for 20 min. Five regions of invaded cells were randomly selected per well. The migration index (%) indicates the number of cells in each group relative to the control.

### Wound healing assay

HPC exosomes with or without the corresponding plasmids were co-inoculated with M0 macrophages in 6-well plates, and the cells were wall-approximated and incubated for 48 h to form a fusion monolayer. Linear wounds were then created with 200 µL sterile plastic tips. Cell debris was washed 1–3 times with phosphate-buffered saline (PBS). Measure the distance the monolayer cells migrated to the wound area at the indicated times. The migration index was [(initial wound width - width measured at the indicated time points)/initial wound width] × 100%. Each experiment was performed at least three times.

### Dual­luciferase reporter assay

Plasmids containing the HOXD9 wild-type 3’UTR sequence and mutant 3’UTR sequence were constructed using pcDNA3.1. Afterward, the plasmids were cloned into luciferase reporter gene vector, Lipofectamine 2000 was added, and transfection complexes containing wild-type and mutant plasmids were prepared separately according to the transfection kit instructions. The transfection complexes were added to the wells containing 293T cells and cultured at 37˚C, 5% CO_2_ for 48 h. Luciferase activity was assayed using a dual luciferase reporter gene assay system (RG027, Beyotime Biotechnology, Shanghai, China), and the ratio of firefly/reninase activity was calculated.

### Chromatin immunoprecipitation (ChIP)-qPCR assay

To begin the process, 1% formaldehyde was added to treat the cells for 10 min at room temperature to cross-link proteins to DNA and stabilize the protein-DNA complex. Following this, the cross-linking reaction was quenched by introducing 125 µM glycine, preventing over-cross-linking and ensuring the preservation of the structural integrity of the complexes. Subsequently, cells were lysed using cell lysis buffer to release chromatin. Chromatin was then sonicated into 200–1000 bp fragments. Afterward, 2 µg of anti-HOXD9 (Santa Cruz Biotechnology; Cat. No sc137134) or anti-mouse IgG (Bitem Biotechnology; Cat. No A7028) antibodies were treated with cross-linked 100 µg DNA (spectrophotometer at 260 nm)/protein immunoprecipitation and incubated at 4˚C for 2 h. In the final step, the protein-DNA complex is eluted from the magnetic beads using elution buffer. Protein-DNA cross-links were disrupted by heat or enzymatic digestion (e.g., proteinase K) to release the DNA. DNA was purified from the samples using a DNA purification kit. q-PCR was performed as described previously. Immunoprecipitated DNA was analyzed on an ABI Prism 7900HT Sequence Detection System.

### Western blot

Cells were collected, lysed using cell lysate containing RIPA, supernatants were aspirated, and cell concentration was determined using a BCA kit. SDS-PAGE gels were prepared, and after protein uploading, electrophoresis was performed. After electrophoresis, the desired proteins were retained according to the experimental requirements, and the proteins were transferred to a PVDF film using semi-dry transfer or wet transfer technique, and then put into 5% BSA or skimmed milk for 2 h to be closed, and incubated with anti-HOXD9 antibody (1:2000) or APOC1 (1:1000) at 4 °C overnight. Strips were removed the next day and the membrane was washed 3 times. Finally, the secondary antibody (1:3000) was incubated for 2 h at room temperature, the membrane was washed and developed.

### Statistical analysis

The experiment was repeated at least three times and the data were expressed as mean ± standard deviation, and unpaired Student’s t-test was used to analyze the differences between the two groups. Differences between multiple groups were analyzed using one-way ANOVA with Tukey’s post-hoc test. *p* < 0.05 (two-tailed) was considered statistically significant. Statistical analysis was performed using GraphPad Prism version 6.0 (GraphPad Software, USA).

## Results

### APOC1 expression is elevated in glomerular podocyte exosomes of DKD

**Fig. 1:**
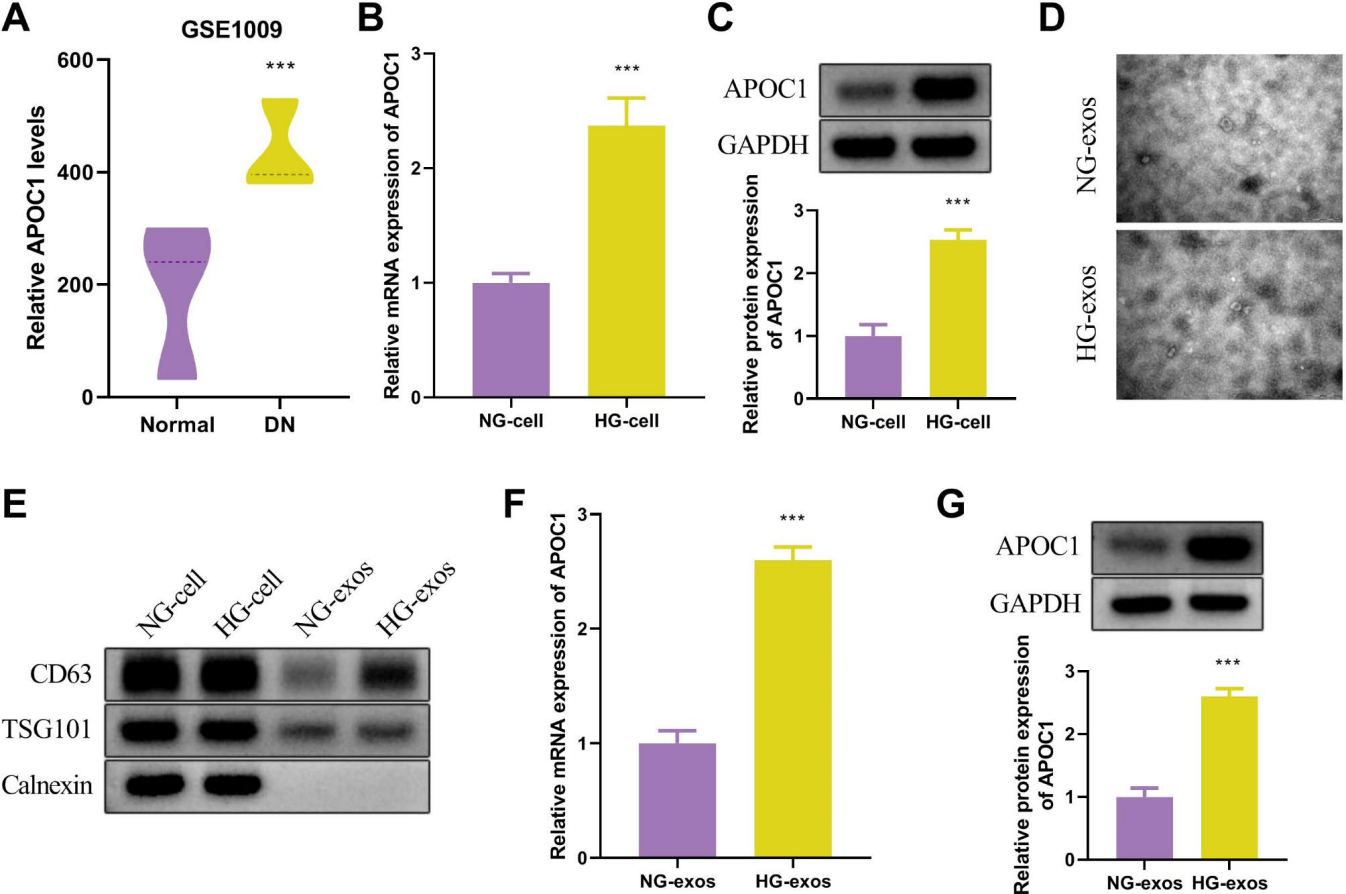
APOC1 expression is elevated in glomerular podocyte exosomes of DKD. (**A**) APOC1 expression analysis in DKD and normal group by GEO database and GSE1009 database. (**B**-**C**) APOC1 expression levels in NG-treated cells and HG-treated cells were analyzed by qRT-PCR and Western blot. (**D**) TEM images of NG-exos and HG-exos. (**E**) Western blot of CD63, TSG101, and Calnexin. (**F**-**G**) APOC1 expression levels in NG-exos and HG-exos were analyzed by qRT-PCR and Western blot. The values in the graphs represent the mean ± SD. * *P* < 0.05; ** *P* < 0.01, *** *P* < 0.001

The expression levels of important genes in diabetes were analyzed by using the DKD microarray dataset GSE1009 in the Gene Expression Database (GEO). The results revealed that protein expression of APOC1 was increased in patients with diabetic kidney disease (Fig. 1A). HPC was utilized in this investigation to create a cell model of diabetic kidney disease to confirm the expression results of APOC1 in DKD. The APOC1 mRNA expression was significantly up-regulated in the high-glucose group compared with the normal glucose-treated group (Fig. 1B). Similarly, the protein expression of APOC1 was measured by Western blot, which was in accordance with the mRNA results, indicating a significant increase in the HG group (Fig. 1C).

Exosomes are considered as possible disease markers since they are linked to various physiopathological processes. Consequently, the expression of APOC1 in podocyte exosomes was further examined in this study. The transmission electron microscopy results in Fig. 1D showed that the morphology and structure of the exosomes in the NG group were expected at 50–100 nm, with a relatively homogeneous size and uniform distribution. Conversely, the exosomes in the HG group were reduced in size compared to the NG group, and the distribution remained nearly unchanged. Furthermore, the exosome marker proteins CD63, TSG101, and endoplasmic reticulum-associated protein Calnexin were detected. The results of CD63, TSG101, and Calnexin was not significantly altered in the HG cell group compared with the NG cell group. In contrast, the protein expression of CD63 and TSG101 increased dramatically in the HG-exos group, and the Calnexin protein was not expressed (Fig. 1E). Additionally, based on the results of mRNA and protein expression of APOC1 in exosomes, APOC1 expression was upregulated in HG-exos group (Fig. 1F and G). The aforementioned findings indicated that exosomes were accurately isolated and purified and the APOC1 expression of HG-exos group was increased.

### APOC1-enriched exosomes originating from HG-treated HPC promoted M1 macrophage activation

**Fig. 2:**
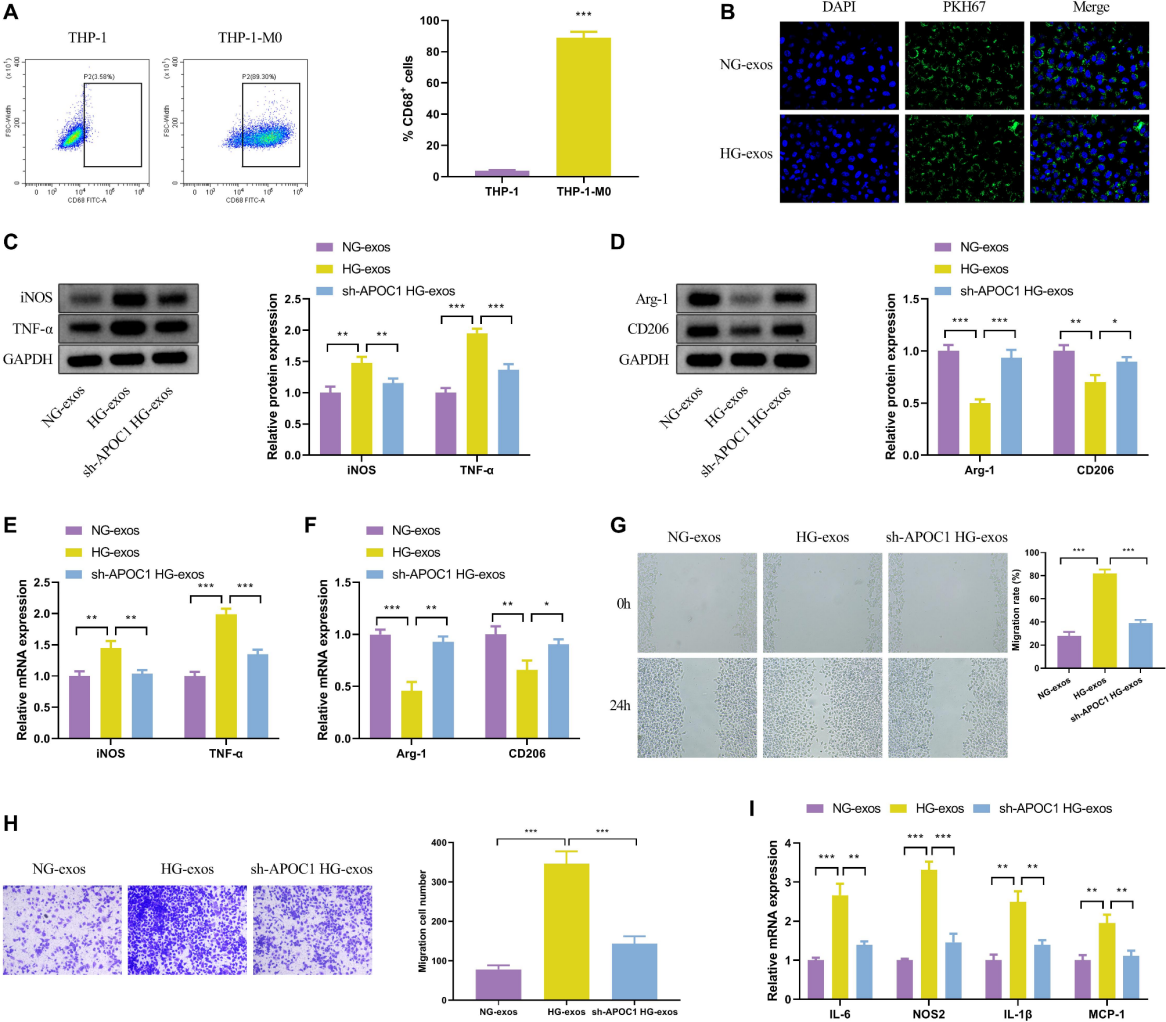
APOC1-enriched exosomes originating from HG-treated HPC promoted M1 macrophage activation. The experiment was divided into NG-exos, HG-exos, and sh-APOC1 + HG-exos groups (**A**) Flow cytometric analysis of THP-1 cells. (**B**) Fluorescence staining of PKH67-labeled HPC exosomes and DAPI-labeled THP-1 M0 cells. (**C**-**D**) Western blot analysis of iNOS, TNF-α, Arg-1, and CD206. (**E**-**F**) The mRNA expressions of iNOS, TNF-α, Arg-1, and CD206. (**G**-**H**) Detection of HPC exosomes and THP-1 M0 cells co-cultured chemotaxis by wound healing assay and Transwell invasion assay. (**I**) The mRNA expressions analysis of IL-6, NOS2, IL-1β, and MCP-1 by qPCR. The values in the graphs represent the mean ± SD. * *P* < 0.05; ** *P* < 0.01, *** *P* < 0.001

To further investigate how APOC1 in exosomes was involved in the process of DKD, this subsection first induced THP-1 into macrophages. The percentage of CD68-positive cells was analyzed by flow cytometry, and the results showed that the percentage of CD68-positive cells was 80%, which was considerably higher compared with the 3% positive cell rate of the control group (Fig. 2A). This evidence demonstrated that THP-1 cells were induced into macrophages. As exosomes can only function if they are successfully taken up by target cells, we labeled the NG-exos and HG-exos groups using PKH67 and then co-cultured them with macrophages, respectively. The nuclei of the macrophages were stained with DAPI to observe the uptake of exosomes by the macrophages. The periphery of macrophages in the NG-exos and HG-exos groups showed green fluorescence, indicating that the exosomes were taken up by macrophages (Fig. 2B).

Moreover, THP-1 and HPC exosomes were co-cultured, in which the control group was kept unchanged and the experimental group was added with high glucose and sh-APOC1 + high glucose treatments, respectively. Compared to the NG-exos group, the results in the HG-exos group presented an increase in the mRNA and protein of iNOS and TNF-α, and a decline in the mRNA and protein of iNOS and TNF-α with the addition of sh-APOC1 (Fig. 2C and E). In contrast to these results, the results of gene and protein expression in M2-type macrophages showed reduced mRNA and protein expression of Arg-1 and CD206 in the HG-exos group and increased expression in the sh-APOC1 HG-exos group (Fig. 2D and F). Therefore, we hypothesized that APOC1 may play a role in macrophage M1-type polarization.

M1-type macrophages promote inflammatory responses, causing elevation of inflammatory factors and release of chemokines. To verify the polarizing effect of APOC1 on M1-type macrophages, the chemotactic function THP-1 using the wound healing assay and the transwell assay were examined. The two parameters demonstrated that the migration rate of the HG-exos group was elevated compared to the NG-exos group, while the sh-APOC1 HG-exos group attenuated the migration rate of the HG-exos group (Fig. 2G and H). Likewise, the qPCR was performed to investigate the secretory function of THP-1. As illustrated in Fig. 2I, the mRNA expression of IL-6, NOS2, IL-1β, and MCP-1 increased in the HG-exos group, consistent with an upregulation of pro-inflammatory cytokines TNF-α and IL-1β, as well as the chemokine MCP-1. Conversely, the expression of all the above mRNAs was significantly reduced in the sh-APOC1 HG-exos group. Overall, the above results signified that the high sugar-treated podocyte exosome APOC1 induced macrophage M1-type polarization.

### HOXD9 modulated exosomal APOC1 derived from HG-stimulated HPC

**Fig. 3:**
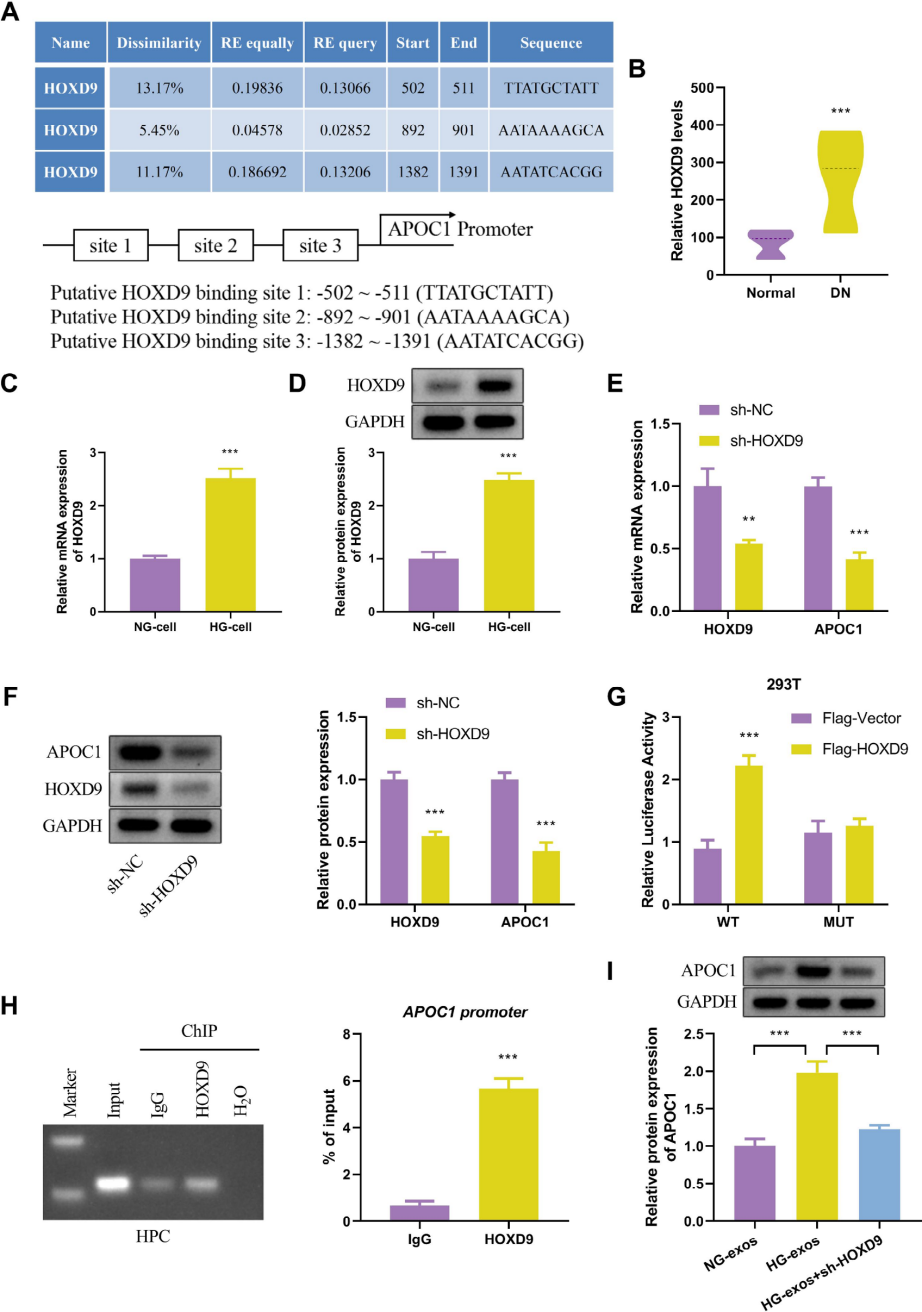
HOXD9 modulated exosomal APOC1 derived from HG-stimulated HPC. (**A**) HOXD9 as a potential transcription factor for APOC1 by PROMO database. (**B**) HOXD9 expression in normal and DN groups based on GSE1009 database. (**C**-**D**) The analysis of HOXD9 in NG-cells and HG-cells by qRT-PCR and Western blot. (**E**-**F**) HOXD9 and APOC1 expression following transfection with or without shHOXD9. (**G**) After co-transfection of HOXD9 and a luciferase reporter gene in HEK293T cells, the activity of APOC1 is detected by luciferase assay. (H) ChIP-PCR was performed to examine the binding of HPXD9 and APOC1. IgG served as a negative control. (**I**) APOC1 expression among NG-exos, HG-exos and HG-exos + sh-HOXD9 groups. The values in the graphs represent the mean ± SD. * *P* < 0.05; ** *P* < 0.01, *** *P* < 0.001

To identify the transcription factors that regulate APOC1, this study predicted the transcription factors of APOC1 using the PROMO database and found HOXD9 to be a potential transcription factor (Fig. 3A). Moreover, we performed a focused analysis of HOXD9 expression using the GSE1009 database, which showed that HOXD9 expression was elevated in DN patients (Fig. 3B). In addition, the findings of Western blotting and qRT-PCR assays were consistent with the database results, which indicated that the mRNA and protein expression levels of HOXD9 were significantly higher in the HG group compared with the NG group (Fig. 3C and D). The results revealed that knockdown of HOXD9 in HPC diminished mRNA levels of HOXD9 and APOC1, and protein expression was likewise reduced (Fig. 3E and F). Additionally, through luciferase reporter assays demonstrated a significant increase in APOC1 promoter activity upon overexpression of HOXD9 (Fig. 3G). The results of ChIP-PCR showed an increased expression of HOXD9 bound to APOC1 in the experimental group compared to the negative control group (IgG) (Fig. 3H). In parallel, the protein expression of APOC1 in high glucose exosomes was observed by knockdown of HOXD9. The results obtained proved that, in contrast to the normal group, APOC1 was elevated in the HG-exos group and restored in the HG-exos + sh-HOXD9 group, indicating that HOXD9 was able to up-regulate APOC1 expression (Fig. 3I). These findings confirmed that HOXD9 was involved in high glucose-induced expression of the exosome APOC1 in podocytes and that HOXD9 may be a positive regulator of APOC1.

### HOXD9/APOC1 axis leads to macrophage M1 polarization through activation of the NF-κB signaling pathway

**Fig. 4:**
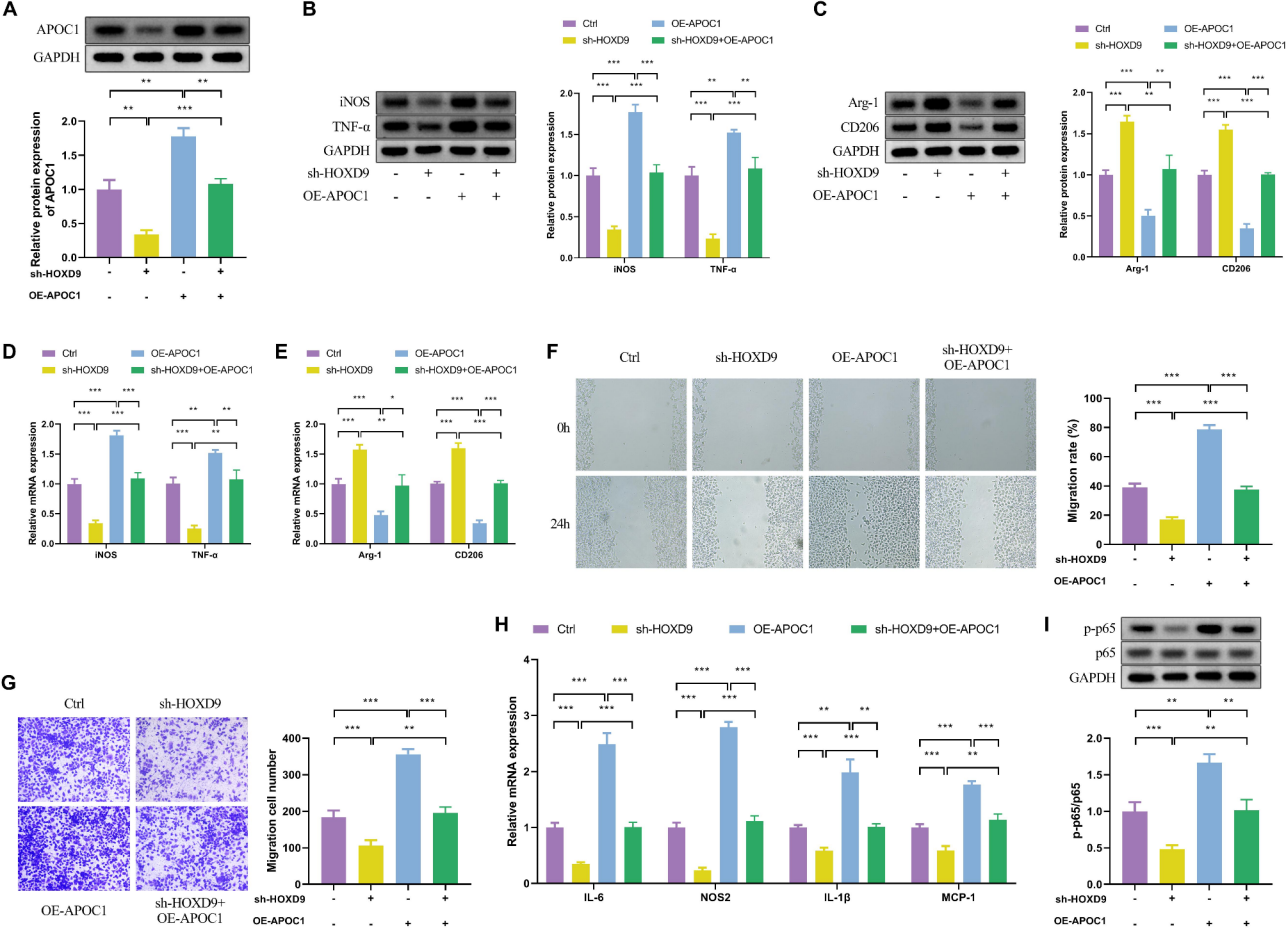
HOXD9/APOC1 axis leads to macrophage M1 polarization through activation of the NF-κB signaling pathway. The experiment was divided into control, sh-HOXD9, OE-APOC1, and sh-HOXD9 + OE-APOC1 groups. (**A**) Western blot of APOC1 expression in HG-exos. (**B**-**E**) The protein and mRNA expressions of iNOS, TNF-α, Arg-1, and CD206 in HPC exosomes and THP-1 M0 co-treatment groups. (**F**-**G**) The chemotaxis of HPC exosomes co-cultured with THP-1 M0 cells was examined by wound-healing assay and transwell invasion assay. (**H**) The mRNA expression analysis of IL-6, NOS2, IL-1β, and MCP-1 by qPCR. (**I**) The detection of p-p65 and p65 expression by western blot. The values in the graphs represent the mean ± SD. * *P* < 0.05; ** *P* < 0.01, *** *P* < 0.001

To further investigate the relationship between HOXD9 and APOC1 and the specific mechanisms causing DKD, the downregulation of HOXD9 and the overexpression of APOC1 in podocytes were simultaneously achieved through the application of lentiviral transfection. The data demonstrated a significant decrease in APOC1 protein expression in the sh-HOXD9 group and an increase in APOC1 protein expression in the OE-APOC1 group as compared to the control group. Upon sh-HOXD9 alongside OE-APOC1, a subtle decline became apparent in contrast to the OE-APOC1 group (Fig. 4A). Moreover, compared with the Control group, the mRNA and protein of iNOS and TNF-α were reduced in the sh-HOXD9 group and significantly augmented in the OE-APOC1 group. The mRNA and protein expression of iNOS and TNF-α in the sh-HOXD9 + OE-APOC1 group were downregulated compared to the OE-APOC1 group (Fig. 4B and D). These implied that HOXD9/APOC1 may lead to the induction of MI-type macrophage polarization. The mRNA and protein expression data of the macrophage-associated factors Arg-1 and CD206 in the M2 type were exactly opposite to the results of iNOS and TNF-α expression (Fig. 4C and E). The wound healing assay and Transwell assay were similar to the above results, which showed the addition of sh-HOXD9 decreased THP-1 cell chemotaxis, while OE-APOC1 accelerated THP-1 chemotaxis (Fig. 4F and G). The above results confirmed that high-glucose induced M1-type polarization of macrophages via HOXD9/APOC1. As shown in Fig. 4H, the mRNA expression of IL-6, NOS2, IL-1β, and MCP-1 were decreased in the sh-HOXD9 group and increased in the OE-APOC1 group. These data indicated that sh-HOXD9 decreased the secretion ability of THP-1 cells and OE-APOC1 accelerated THP-1 secretion.

Activation of NF-κB signaling pathway is one of the key drivers of M1 macrophage polarization, and the phosphorylation level of p65 often serves as a marker of NF-κB signaling pathway activation [27]. Therefore, p65 and p-p65 protein levels were detected, and it was found that p65 phosphorylation level was decreased in the sh-HOXD9 group and OE-APOC1 group had elevated p-p65 protein expression, and p65 phosphorylation was downregulated in the sh-HOXD9 + OE-APOC1 group compared with the OE-APOC1 group (Fig. 4I). In summary, the HOXD9/APOC1 axis exacerbated the progression of DKD by activating the NF-κB signaling pathway leading to macrophage M1-type polarization.

### Knocking down APOC1 enhances the inhibitory effect of liraglutide on macrophage M1 polarization

**Fig. 5:**
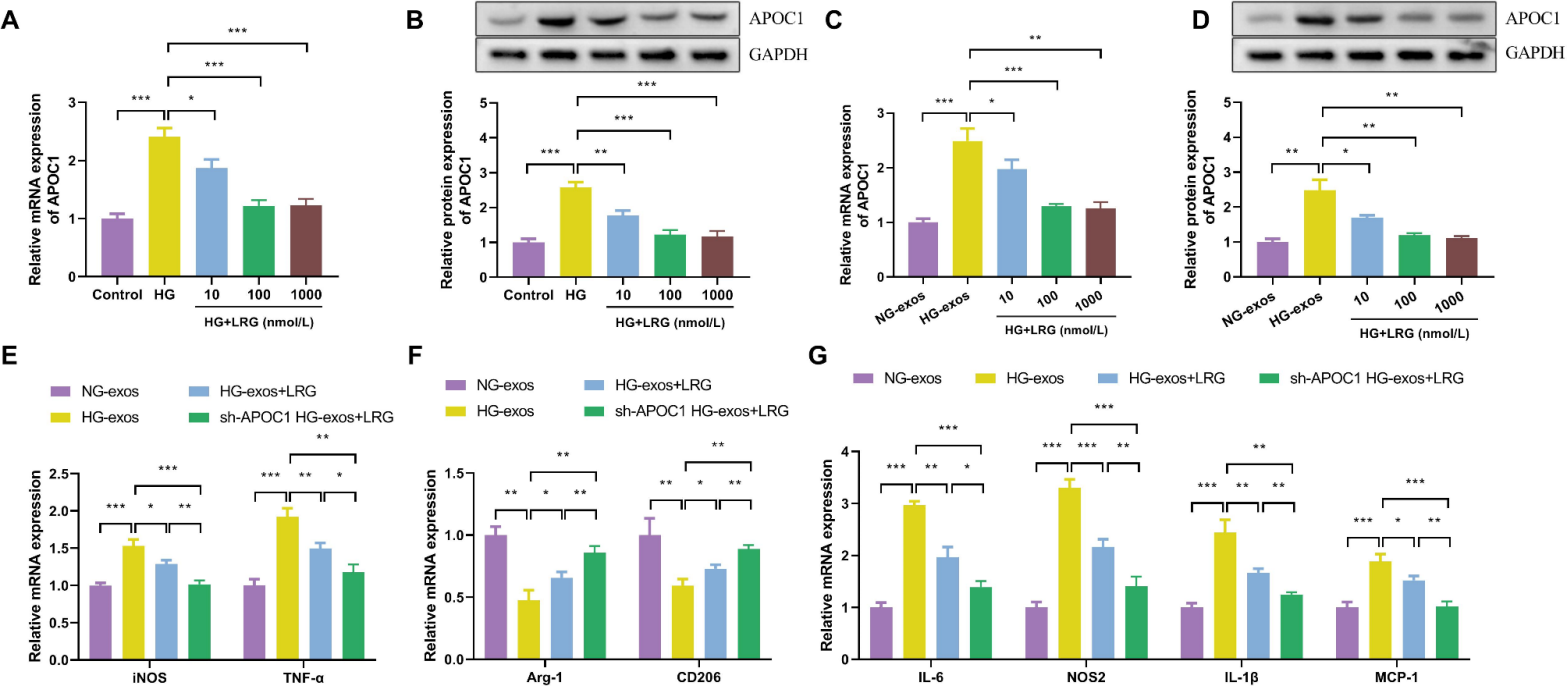
Knocking down APOC1 enhances the inhibitory effect of liraglutide on macrophage M1 polarization. (**A**-**B**) APOC1 expression levels in NG-treated cells and HG-treated cells were analyzed by qRT-PCR and Western blot. (**C**-**D**) APOC1 expression levels in NG-exos and HG-exos were analyzed by qRT-PCR and Western blot. (**E-F**) The mRNA expressions of iNOS, TNF-α, Arg-1, and CD206. (**G**) The mRNA expressions analysis of IL-6, NOS2, IL-1β, and MCP-1 by qPCR. The values in the graphs represent the mean ± SD. * *P* < 0.05; ** *P* < 0.01, *** *P* < 0.001

Liraglutide (LRG), a GLP-1 receptor agonist, has been investigated for its potential therapeutic effects on DKD [25]. However, there have been no studies on LRG and APOC1. To determine whether LRG exerts its effects through the modulation of APOC1, we first examined its regulatory role on APOC expression in podocytes under high-glucose conditions. Compared to the Control group, the mRNA and protein levels of APOC1 in podocytes were significantly elevated under high-glucose conditions, consistent with the results presented in Fig. 1B and C (Fig. 5A and B). This aberrant expression of APOC1 was markedly ameliorated following LRG intervention. Notably, a more pronounced improvement was observed at higher concentrations of LRG (100 nmol/L and 1000 nmol/L) compared to HG + LRG (10nmol/L) (Fig. 5A and B). Subsequently, we isolated extracellular vesicles from podocytes in each group and assessed the expression of APOC1 within these vesicles. The expression of APOC1 mRNA and protein in the extracellular vesicles indicated that its upregulation in the HG-exos group was reversed by LRG intervention (Fig. 5C and D). However, at a concentration of 100nmol/L, the expression level of APOC1 plateaued, and no significant difference was observed in the APOC1 expression levels between the podocyte extracellular vesicles treated with 100nmol/L and 1000nmol/L of LRG. The comparison between these two groups revealed no statistically significant difference (*P* > 0.05). Therefore, we selected a LRG concentration of 100nmol/L for a 24-hour treatment in subsequent experiments (Fig. 5C and D).

In order to elucidate the role of APOC1 in the modulation of macrophage M1 polarization by LRG, we conducted co-cultures of THP-1 cells with podocyte-derived extracellular vesicles that had been subjected to high-glucose conditions in the presence of LRG. qRT-PCR assessment of markers indicative of macrophage polarization demonstrated that THP-1 cells co-cultured with HG-exos displayed elevated expression of M1 macrophage markers, specifically iNOS and TNF-α, and reduced expression of M2 macrophage markers, namely Arg-1 and CD206, when compared to those co-cultured with NG-exos. The intervention with LRG led to a significant reversal of this polarization pattern. Moreover, the downregulation of APOC1 in podocyte extracellular vesicles was found to potentiate the inhibitory effect of LRG on macrophage M1 polarization, as depicted in Fig. 5E and F. In alignment with this, the suppression of APOC1 further enhanced the ability of LRG to reduce the secretion of inflammatory cytokines by macrophages, including IL-6, NOS2, IL-1β, and MCP-1. These findings collectively underscore the potential of APOC1 knockdown to amplify the inhibitory impact of LRG on macrophage M1 polarization.

## Discussion

DKD, as one of the most common microvascular complications of diabetes, has been reported in many studies. Nevertheless, the prevention and treatment of DN are not well established. Ma et al. reported that baicalein (BAI) increased renal glutathione peroxidase (GSH-PX), superoxide dismutase (SOD), and catalase (CAT) levels, as well as lowered malondialdehyde (MDA) levels, and inhibited secretion of inflammatory factors. Further investigation of the mechanism revealed that BAI attenuated oxidative stress and inflammation in diabetic nephropathy through Nrf2 and MAPK signaling pathways [28]. Mitochondrial damage and ferroptosis occur in DKD. Previous study observed that NAC enhanced mitochondrial GSH activity and down-regulated SOD_2_ acetylation. They demonstrated that NAC sustained mitochondrial redox homeostasis and diminished iron death in HG-exposed MDCK cells, offering protection in diabetic kidney disease [29]. Therefore, a high-glucose model using HPC was established in this study to delve into the specific mechanisms of diabetic kidney disease.

Given that APOC1 plays a pivotal role in mediating inflammatory responses and in light of diabetic kidney disease being intricately associated with inflammation, the salience of APOC1 in the progression of diabetic kidney disease is made apparent. In this experiment, the expression of APOC1 was first measured, in line with previous findings, the database and in vitro experiments shown that APOC1 expression was increased in DKD patients. An exosome is a nanoscale vesicle for intercellular communication that can also participate in inflammatory and metabolic responses leading to disease [30]. Our findings extracted and isolated HPC exosomes and revealed that APOC1 was similarly elevated within exosomes. We further investigated the effect of APOC1 in exosomes on macrophages. As expected, APOC1 caused macrophage M1 polarisation, leading to an increase in pro-inflammatory factors such as iNOS, TNF-α, and IL-1β, and chemokines MCP-1. Upon knockdown of APOC1, contrary to these results, macrophages were converted to M2 type, attenuating the inflammatory response. Peng et al. found that intrahepatic macrophage reprogramming in acute-on-chronic liver failure (ACLF) was associated with lipid metabolism, and among the lipid-associated genes were APOC1 [31, 32]. In addition, another research reported that hepatic macrophages in *Schistosoma mansoni* infection, suggesting that macrophages can regulate systemic lipid and glucose metabolism [33]. Similar to our study, we tentatively hypothesized that APOC1 induces macrophage M1-type polarization.

Homeobox (HOX) genes are transcription factors in embryonic development and cell differentiation. Homeobox D9 (HOXD9) is one of several HOXD genes located in the region of chromosome 2q31-2q37 [34]. In this study, HOXD9 was predicted as a potential transcription factor for APOC1 using the PROMO database and GSE1009. Recent investigations have noticed that HOXD9 is abnormally expressed in many cancers, such as gastric cancer, hepatocellular carcinoma, and thyroid cancer [35]. However, few studies have been reported on HOXD9 in diabetic kidney disease. Based on the database results, HOXD9 was knocked down to verify its effect on APOC1 in this research. Western blot results demonstrated that in HPC, HOXD9 was augmented in the high glucose group, and APOC1 expression was reduced as HOXD9 levels decreased. Likewise, the same function of HOXD9 was seen in podocyte exosomes. Studies indicated that down-regulation of HOXD9 inhibited the proliferation, migration, and invasion of mesenchymal thyroid cancer (ATC) cells [36]. In addition, another observation elucidate that knockdown of HOXD9 in vitro effectively inhibited the proliferation, migration, and invasion of gastric cancer (GC) cells. HOXD9 is likely to affect GC cell proliferation by activating the TGF-β/Smad signaling pathway [37].

APOC1 functions as an immunological biomarker exerting influence on macrophage polarization [14]. Here, we confirmed the role of the HPC exosomes HOXD9 and APOC1 in macrophage polarization by knockdown of HOXD9 and overexpression of APOC1. The research suggested that HOXD9 is an upstream transcription factor of APOC1 and that HOXD9 positively regulated APOC1. Interestingly, a recent study explored the role of homology cassette D9 (HOXD9) and sodium channel epithelial cell 1α subunit (SCNN1A) in pancreatic cancer (PC) progression. The results showed that HOXD9 activated the transcription of SCNN1A and promoted the malignant development of PC [38]. In line with our findings, another report examined the interaction mechanism between APOC1 and mitochondrial carrier homolog 2 (MTCH2) in osteosarcoma. The evidence indicated significant upregulation of APOC1 and MTCH2 expression in osteosarcoma SAOS-2 cells. Up-regulation of MTCH2 restored the elevated oxidative phosphorylation and reduced the Warburg effect caused by APOC1 silencing [39]. Elevated expression levels of iNOS and TNF-α usually represent the formation of M1-type macrophages, which promote inflammation, increased chemokine secretion, and enhanced cellular mobility. Similarly, elevated Arg-1 and CD206 indicate enhanced cellular anti-inflammatory function and conversion of macrophages to M2-type macrophages [40, 41]. The results of the present study manifested that HOXD9 and APOC1 work together to cause macrophage polarization into the M1 type.

The regulation of macrophage M1 polarization by the NF-κB signaling pathway has been extensively described. For example, Lin and colleagues illustrated that IL-26 expression was increased in the synovium of rheumatoid arthritis and that IL-26 promoted macrophage differentiation from CD80^+^ M1 macrophages, concomitantly activating the NF-κB pathway [42]. More than that, another study reported increased CX3CL1 in ankylosing spondylitis, leading to elevated expression of M1-type macrophage markers and inflammatory factors that promote osteoclast differentiation. Subsequently, they found that blocking the NF-κB pathway prevented M1-type macrophage polarization, which in turn diminished the level of inflammation and depressed osteoclast differentiation, suggesting that CX3CL1 induced macrophage M1 polarization through the NF-κB pathway [43]. In agreement with previous findings, our experiment confirmed that HOXD9/APOC1 axis evoked macrophage M1 polarization via the NF-κB signaling pathway.

Investigations into the impact of liraglutide on macrophage polarization are burgeoning in the scientific community. A wealth of evidence suggests that liraglutide has the capacity to curb the pro-inflammatory polarization (M1) of macrophages, nudging them towards an anti-inflammatory phenotype (M2) [44]. This pivotal shift is instrumental in dampening inflammatory reactions and ameliorating tissue damage. Notably, recent studies have, in a pioneering move, exposed the anti-inflammatory and renoprotective properties of liraglutide within a Type 1 Diabetes Mellitus (T1DM) model, which are underpinned by alterations in macrophage polarization [45]. More specifically, liraglutide has been shown to diminish the prevalence of M1 macrophages, consequently lowering the M1 to M2 ratio—a critical development in the context of kidney injury related to diabetes [46]. Such a transformation is beneficial in reducing the kidney’s inflammatory burden, presenting a fresh avenue for the management of diabetic nephropathy. Nonetheless, the extent to which liraglutide’s effects are mediated through APOC1 awaits further elucidation. In the present investigation, we observed that liraglutide effectively inhibits the polarization of macrophages towards the M1 phenotype and subsequently mitigates inflammation. In this study, we have made a novel discovery that the suppression of APOC1 potentiates the inhibitory effect of liraglutide on macrophage M1 polarization, which is a significant advancement over previous research. This finding aligns with the theory that both APOC1 and liraglutide exert their effects by influencing the polarization state of macrophages.

However, we have specifically addressed the regulatory impacts of APOC1 and HOXD9 on podocytes and podocyte-derived exosomes under in vitro conditions. Therefore, further in vivo animal experiments are needed to support the present conclusions. In addition, How liraglutide regulates the expression of APOC1 is one of the future research directions.

Taken together, the above findings argued that HOXD9/APOC1 expression was elevated in exosomes of high glucose-treated podocytes, causing macrophage M1-type polarization through the NF-κB signaling pathway. The present study elucidated that APOC1 may be a target of liraglutide for DKD.

## Electronic supplementary material

Below is the link to the electronic supplementary material.

Supplementary Material 1

## Data Availability

Data is available from the corresponding author upon reasonable request.
