# Supplementary figures and images for: HOXD9/APOC1 axis promotes macrophage M1 polarization to exacerbate diabetic kidney disease progression through activating NF-κB signaling pathway
Source: Hereditas. 2024 Nov 7;161:40. doi: 10.1186/s41065-024-00345-9 (PMC11542400; doi:10.1186/s41065-024-00345-9)

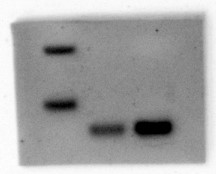


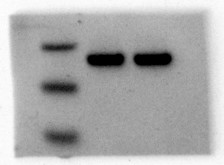


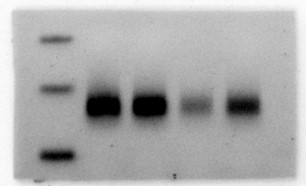


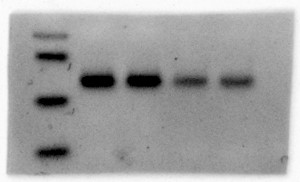


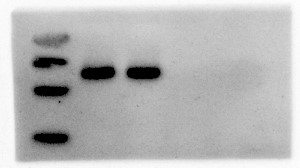


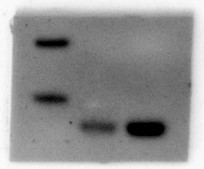


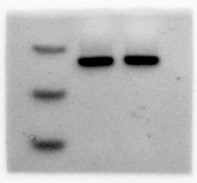


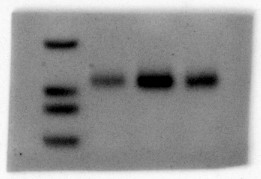


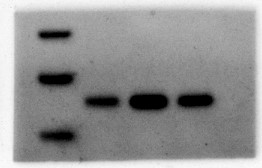


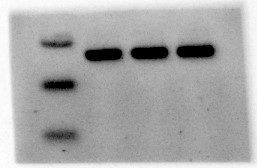


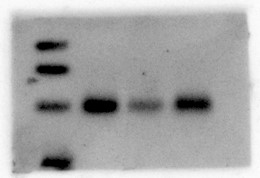


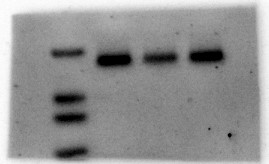


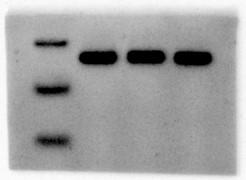


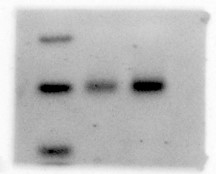


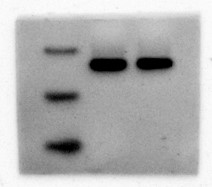


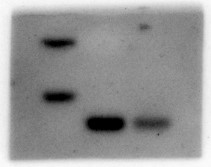


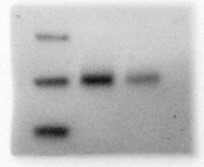


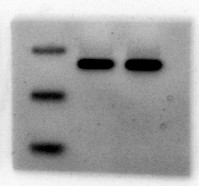


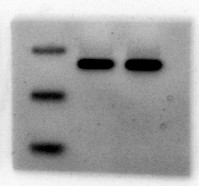


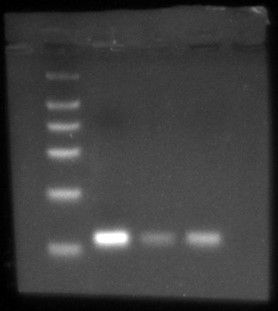


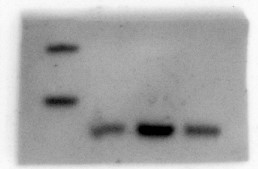


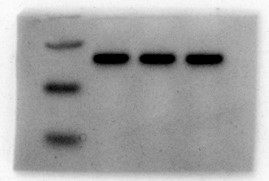


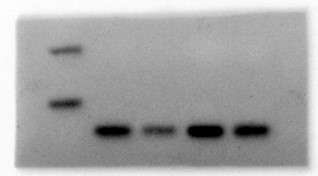


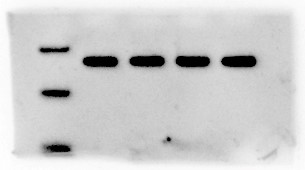


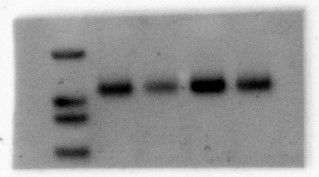


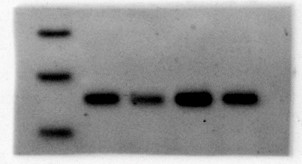


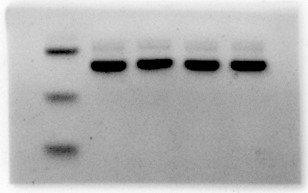


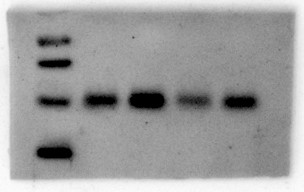


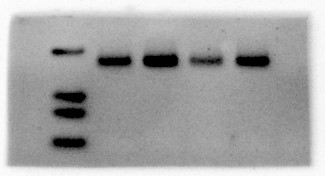


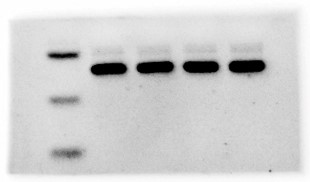


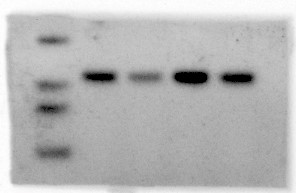


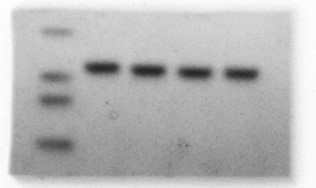


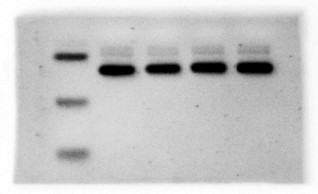


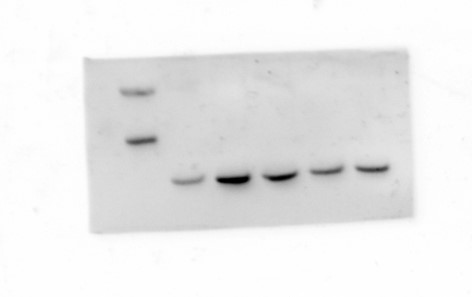


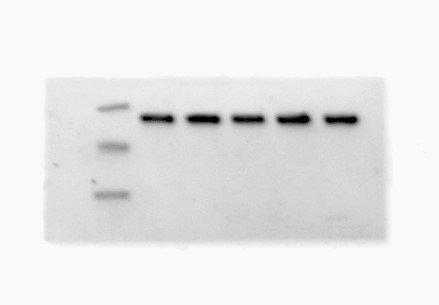


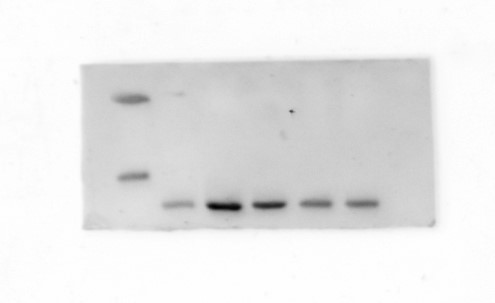


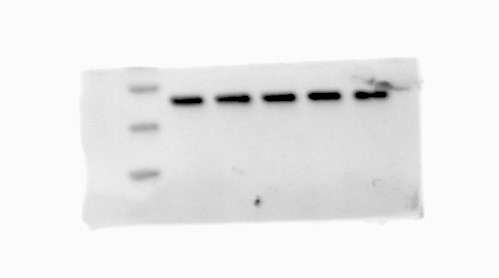

Supplement: Supplementary file 1 — Supplementary Material 1 [file 41065_2024_345_MOESM1_ESM.docx]
